# Association between arterial stiffness and orthostatic hypotension: A systematic review and meta-analysis

**DOI:** 10.3389/fphys.2023.1164519

**Published:** 2023-04-28

**Authors:** Alicia Saz-Lara, Iván Cavero-Redondo, Vicente Martínez-Vizcaíno, Maribel Lucerón-Lucas-Torres, Carlos Pascual-Morena, Irese Sequí-Domínguez

**Affiliations:** ^1^ Universidad de Castilla-La Mancha, Health and Social Research Center, Cuenca, Spain; ^2^ Universidad Autónoma de Chile, Facultad de Ciencias de la Salud, Talca, Chile

**Keywords:** orthostatic hypotension, arterial stiffness, pulse wave velocity, adults, meta-analysis

## Abstract

**Background:** Orthostatic hypotension, defined as a decrease in blood pressure on standing, is associated with an increased risk of mortality and cardiovascular events in the general population. In addition, it has recently been suggested that arterial stiffness is independently associated with orthostatic hypotension, which may be due to a loss of the buffering effect of the ascending aorta and an early return of pressure waves. However, the specific mechanisms underlying this association remain unclear. Thus, we aimed to evaluate the association between orthostatic hypotension and arterial stiffness in the adult population.

**Methods:** PubMed, Scopus, Web of Science, and Cochrane Library databases were searched from inception to 31 January 2022. The DerSimonian and Laird method was used to calculate pooled odds ratio (OR) estimates and their respective 95% confidence intervals (95% CI) for the association between orthostatic hypotension and arterial stiffness.

**Results:** Overall, 11 studies were included, with a total of 10,611 subjects. Our results showed that increased arterial stiffness raises the risk of orthostatic hypotension (OR: 1.40, 95% CI: 1.28–1.54), with a stronger association at central arterial stiffness (OR: 1.50, 95% CI: 1.34–1.68) than at peripheral arterial stiffness (OR: 1.29, 95% CI: 1.17–1.43).

**Conclusion:** Our findings showed that increased arterial stiffness raises the risk of orthostatic hypotension by 40% among the adult population. Considering that orthostatic hypotension, which is usually a consequence of antihypertensive treatment, has been widely associated with the risk of cardiovascular events, appropriate control of arterial stiffness could be a clinical strategy to prevent cardiovascular morbidity and mortality.

## 1 Introduction

Orthostatic hypotension (OH) is a condition of increasing interest in scientific research. Certain neurological diseases are associated with OH; however, OH can also occur due to non-neurological causes. OH is defined by consensus as a decrease in systolic blood pressure by at least 20 mmHg or a decrease in diastolic blood pressure by at least 10 mmHg within the first 3 min of standing upright (Freeman et al., 2011). When the upright posture is adopted, the baroreceptors located at the level of the carotid artery and the aorta are activated and a decrease in blood pressure is produced, causing inhibition of the parasympathetic nervous system and activation of the sympathetic nervous system. This activation leads to an increase in blood levels of noradrenaline, adrenaline, and plasma renin activity, thus raising peripheral arterial resistance and cardiac output to maintain blood pressure (Shibao et al., 2013).

This physiological mechanism may interfere with the presence of atheromatous plaques by sustained high blood pressure levels or by the action of various toxicants acting on the endothelium, leading to sustained arterial hypertension and, thus, favoring an abnormal blood pressure response with orthostasis. Even in subjects without baseline hypertension, OH is associated with increased arterial stiffness, (Takahashi et al., 2015), as measured by pulse wave velocity (PWv). The association is greater when PWv is higher (Mattace-Raso et al., 2006). Arterial stiffness is inversely associated with baroreceptor sensitivity; in these subjects, the mechanisms of blood pressure regulation may not be triggered by the postural change since the activity of the baroreceptor system is diminished by arterial stiffness, thus favoring the fall in blood pressure after orthostasis (Okada et al., 2012).

However, there is limited evidence supporting an association between OH and arterial stiffness, and no meta-analysis has amalgamated this evidence or synthesized this relationship. Thus, the purpose of this systematic review and meta-analysis was to provide a synthesis of the evidence regarding the relationship between OH and arterial stiffness, as measured by its reference standard, PWv.

## 2 Methods

This study, a systematic review and meta-analysis, was performed according to the Meta-analysis of Observational Studies in Epidemiology (MOOSE) statement (Stroup et al., 2008) and was conducted following the recommendations of the Cochrane Collaboration Handbook (Higgins and Green, 2011). This study was registered in PROSPERO (registration number: CRD42022304329).

### 2.1 Search strategy

A systematic search was conducted independently by two reviewers (I.C.-R. and A.S.-L.) and was performed through four databases, namely, Scopus, PubMed, Cochrane Library, and Web of Science, from their inception to 13 March 2022. To perform the search, the following free terms combined with Boolean operators were used, following the PICO (population, intervention/exposure, comparison, and outcome) strategy: “Adults,” “Adult population,” “Adult subjects,” “Arterial stiffness,” “Pulse wave velocity,” “PWv,” “Aortic stiffness,” and “Orthostatic hypotension” (Supplementary Table S1). Furthermore, we searched previous systematic reviews or meta-analyses, and the references of the included articles.

### 2.2 Study selection

Studies on the association between OH and arterial stiffness were included in this systematic review and meta-analysis. The inclusion criteria were as follows: i) population: adult subjects with pathologies; ii) exposure: arterial stiffness measured by PWv; iii) comparison: subjects with OH versus subjects without OH; and iv) outcome: OH defined as a decrease in SBP of at least 20 mmHg and/or DBP of at least 10 mmHg. We excluded i) review articles, editorials, or case reports; ii) studies that did not include cross-sectional data on the association between OH and arterial stiffness; and iii) articles that were not written in English or Spanish.

### 2.3 Data extraction and quality assessment

Study selection, data extraction, and quality assessment of the studies were performed independently by two researchers (I.C.-R. and A.S.-L.), excluding those studies that did not meet the eligibility criteria. Disagreements were resolved by consensus or with the intervention of a third researcher (V.M.-V.).

Table 1 shows the main characteristics of the included studies and covers information on the following: (1) reference: first author and year of publication; (2) country in which the study data were collected; (3) study design (cross-sectional analysis of cohort studies or cross-sectional studies); (4) population characteristics: sample size (%female), mean age, pathologies prevalence in OH (hypertension, diabetes mellitus, heart failure, myocardial infarction, etc.); (5) exposure (arterial stiffness): PWv index (brachial to ankle PWv [ba-PWv] and carotid to femoral PWv [cf.-PWv]), device, baseline PWv levels; and (6) outcome (OH): assessment points (after standing) and OH prevalence.

**TABLE 1:** Characteristics of the included studies in the systematic review and meta-analysis.

Reference	Country	Study design	Population characteristics			Exposure: Arterial stiffness			Outcome: Orthostatic hypotension	
			Sample size (n, %female)	Mean age (years)	Pathology prevalence in OH (%)	PWv index	Device	Basal PWv levels (m/s)	Assessment points	OH prevalence (%)
Mattace-Raso et al. (2006)	Netherlands	Cross-sectional analyses from a cohort	OH: 724 (58.8)	OH: 73.4 ± 7.1	HT: 23.9	cf-PWv	Complior	OH: 14.2 ± 3.3	1, 3, and 5 min after standing	21.5
			NOH: 2638 (57.4)	NOH: 71.6 ± 6.5	DM: 8.1			NOH: 13.3 ± 2.9		
Protogerou et al. (2008)	Greece	Cross-sectional analyses from a cohort	69	53.4 ± 8.8	HT: 100.0	cf-PWv	Complior	10.3 ± 2.1	1 min after standing	13.0
					DM: 8.7					
					CVD: 7.3					
Aso et al. (2012)	Japan	Cross-sectional	OH: 30 (50.0)	OH: 60.2 ± 11.3	HT: 60.0	ba-PWv	PWV/ABI	OH: 19.1 ± 5.0	1, 2, and 3 min after standing	28.6
			NOH: 75 (53.3)	NOH: 58.0 ± 12.9	DM: 100.0			NOH: 16.3 ± 3.8		
Lu et al. (2014)	Taiwan	Cross-sectional analyses from a cohort	OH: 59 (26.9)	OH: 68.1 ± 15.5	HT: 76.3	cf-PWv	VP-2000	OH: 14.3 ± 6.1	3 min after standing	13.8
					DM: 35.6					
					CVA: 8.5					
			NOH: 370 (23.5)	NOH: 64.6 ± 16.5	CAD: 39.0			NOH: 11.6 ± 4.4		
					HF: 35.6					
					MI: 13.6					
Meng et al. (2014)	China	Cross-sectional	OH: 49 (55.1)	OH: 68.0 ± 7.4	HT: 40.8	ba-PWv	VP1000	OH: 20.5 ± 4.5	30 s and 2 min after standing	4.9
			NOH: 961 (58.0)	NOH: 64.6 ± 7.7	DM: 12.2			NOH: 17.3 ± 3.7		
Sung et al. (2014)	Taiwan	Cross-sectional analyses from a cohort	OH: 100 (20.0)	OH: 68.0 ± 16.0	HT: 75.0	ba-PWv	VP-2000	OH: 14.5 ± 4.2	3 min after standing	16.3
					DM: 34.0			NOH: 12.9 ± 3.2		
					CAD: 31.0			OH: 13.8 ± 5.4		
			NOH: 513 (25.0)	NOH: 64.0 ± 17.0	Stroke: 10.0	cf-PWv		NOH: 11.6 ± 4.5		
					HF: 32.0					
Liu et al. (2016)	China	Cross-sectional	OH: 61 (52.5)	OH: 68.4 ± 7.5	HT: 44.3	ba-PWv	VP1000	OH: 20.2 ± 4.9	0 and 2 min after standing	5.6
			NOH: 1038 (58.2)	NOH: 64.4 ± 7.8	DM: 14.8			NOH: 17.1 ± 3.7		
Chi et al. (2019)	China	Cross-sectional analyses from a cohort	OH: 461 (30.6)	OH: 68.0 ± 5.9	HT: 50.7	ba-PWv	BP-203RPE III	OH: 17.5 (15.6-19.7)	1 and 3 min after standing	25.4
			NOH: 1357 (33.4)	NOH: 66.5 ± 5.6	DM: 20.6			NOH: 16.4 (14.7-17.9)		
Cremer et al. (2020)	France	Cross-sectional	OH: 210 (50.5%)	OH: 80.3 ± 3.8	HT: 65.9	cf-PWv	SphygmoCor	OH: 15.5 ± 3.5	1, 2, and 3 min after standing	18.0
					DM: 10.6					
					Dyslipidemia: 41.3					
					Stroke: 0.5					
			NOH: 942 (62.2)	NOH: 80.0 ± 3.8	MI: 1.4			NOH: 14.5 ± 3.2		
					HF: 1.4					
					Cancer: 11.9					
Kirkham et al. (2020)	United Kingdom	Cross-sectional analyses from a cohort	OH: 23 (26.0%)	OH: 72.2 ± 10.3	HT: 87.0	cf-PWv	Complior	OH: 15.2 ± 3.8	3 min after standing	15.8
			NOH: 123 (24.0)	NOH: 68.0 ± 11.5	DM: 26.0			NOH: 12.7 ± 2.6		
					CKD: 100.0					
Li et al. (2021)	China	Cross-sectional	OH: 40 (60.0%)	OH: 61.3 ± 10.6	HT: 77.5	ba-PWv	VP1000	OH: 19.6 ± 4.0	1 and 3 min after standing	6.3
			NOH: 590 (61.0)	NOH: 56.4 ± 10.2	DM: 20.0			NOH: 16.8 ± 3.6		

Data are shown as mean ± standard deviation (SD) or interquartile range; ba-PWv, brachial to ankle pulse wave velocity; CAD, coronary artery diseases; cf-PWv, carotid to femoral pulse wave velocity; CKD, chronic kidney disease; CVA, cerebral vascular accident; CVD, cardiovascular disease; DM, diabetes mellitus; HF, heart failure; HT, hypertension; MI, myocardial infarction; and OH, orthostatic hypotension.

The quality assessment tool for observational cohort and cross-sectional studies from the United States National Institute of Health National Heart, Lung, and Blood Institute (National Institutes of Health, 2022) was used to assess the risk of bias according to the following domains: quality of the research question, reporting of the population definition, participation rate, recruitment, sample size, appropriateness of statistical analyses, timeframe for associations, exposure levels, ascertainment of the exposure, appropriateness of the outcome measured, outcome blinding of researchers, loss to follow-up, and confounding variables. The overall bias of each study was considered “good” if most criteria were met and there was a low risk of bias, “fair” if some criteria were met and there was a moderate risk of bias, or “poor” if few criteria were met and there was a high risk of bias.

### 2.4 Data synthesis and statistical analysis

The DerSimonian and Laird random-effects method (DerSimonian and Kacker, 2007) was used to compute pooled estimates of odds ratios (ORs) and their respective 95% confidence intervals (95% CIs) for the association between OH and arterial stiffness. In addition, the association between OH and central and peripheral AS was evaluated. Meta-analyses required at least five studies in each exposure group. (Jackson and Turner, 2017). Heterogeneity was examined using the *I* ^2^ statistic (Higgins and Thompson, 2002), which ranges from 0% to 100%. According to *I* ^2^ values, heterogeneity was considered not important (0%–30%), moderate (30%–60%), substantial (60%–75%), or considerable (75%–100%). The corresponding *p*-values were also considered.

Sensitivity analysis (systematic reanalysis removing studies one at a time) was conducted to assess the robustness of the summary estimates. Subgroup analyses were performed according to mean age (<65 or >65 years). Random effects meta-regression analyses addressed whether mean age, percentage of female subjects, smoking history, hypertension, and diabetes mellitus prevalence modified the association between OR and arterial stiffness. Finally, publication bias was assessed using Egger’s regression asymmetry test (Sterne et al., 2001) using a level <0.10 to determine whether publication bias might be present.

All statistical analyses were conducted with Stata SE software, version 15 (StataCorp, College Station, TX, United States).

## 3 Results

### 3.1 Baseline characteristics

**FIGURE 1:**
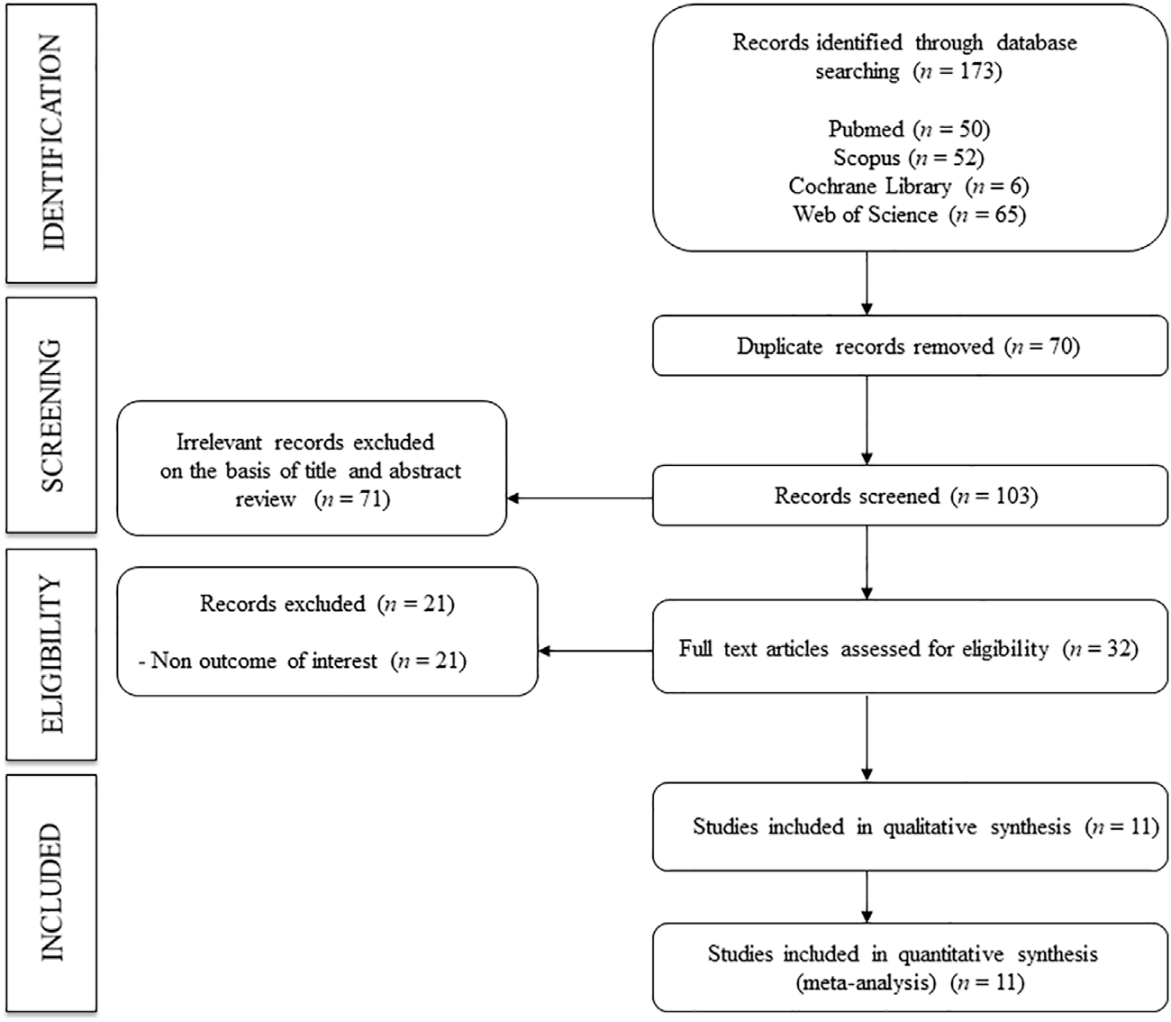
Flowchart: search strategy.

In total, 11 studies (Mattace-Raso et al., 2006; Protogerou et al., 2008; Aso et al., 2012; Lu et al., 2014; Meng et al., 2014; Sung et al., 2014; Liu et al., 2016; Chi et al., 2019; Cremer et al., 2020; Kirkham et al., 2020; Li et al., 2021) were included in the systematic review and meta-analysis (Figure 1). Of the included studies, six were prospective longitudinal studies, (Mattace-Raso et al., 2006; Protogerou et al., 2008; Lu et al., 2014; Sung et al., 2014; Chi et al., 2019; Kirkham et al., 2020), and five were cross-sectional studies. (Aso et al., 2012; Meng et al., 2014; Liu et al., 2016; Cremer et al., 2020; Li et al., 2021). Studies were conducted in seven countries: four in China (Meng et al., 2014; Liu et al., 2016; Chi et al., 2019; Li et al., 2021), two in Taiwan (Lu et al., 2014; Sung et al., 2014), and one each in the Netherlands (Mattace-Raso et al., 2006), Greece (Protogerou et al., 2008), Japan (Aso et al., 2012), France (Cremer et al., 2020), and the United Kingdom. (Kirkham et al., 2020). Records were published between 2006 and 2020 and included a total of 10,611 subjects (aged 37.0–80.0 years). Regarding the type of exposure, different methods were used to measure PWv: six samples for ba-PWv (Aso et al., 2012; Meng et al., 2014; Sung et al., 2014; Liu et al., 2016; Chi et al., 2019; Li et al., 2021) and six samples for cf.-PWv. (Mattace-Raso et al., 2006; Protogerou et al., 2008; Lu et al., 2014; Sung et al., 2014; Cremer et al., 2020; Kirkham et al., 2020). Finally, the measurement of OH was performed in the range of 30 s to 5 min after standing.

### 3.2 Quality assessment and potential bias

The overall risk of bias for studies examining the association between OH and arterial stiffness was fair in 45.5% of the studies and poor in 54.5% (Supplementary Table S2). For all exposures, we were able to identify three main reasons for a poor risk of bias: (i) the follow-up time was not reported or was not long enough to establish an association between the exposure and outcome; (ii) the participation rate of eligible persons was not reported; and (iii) a sample size justification was not reported. In addition, due to the cross-sectional design of the studies, none of the studies provided information on whether the researchers were blinded to the exposure status of the participants.

### 3.3 Association between orthostatic hypotension and arterial stiffness

**FIGURE 2:**
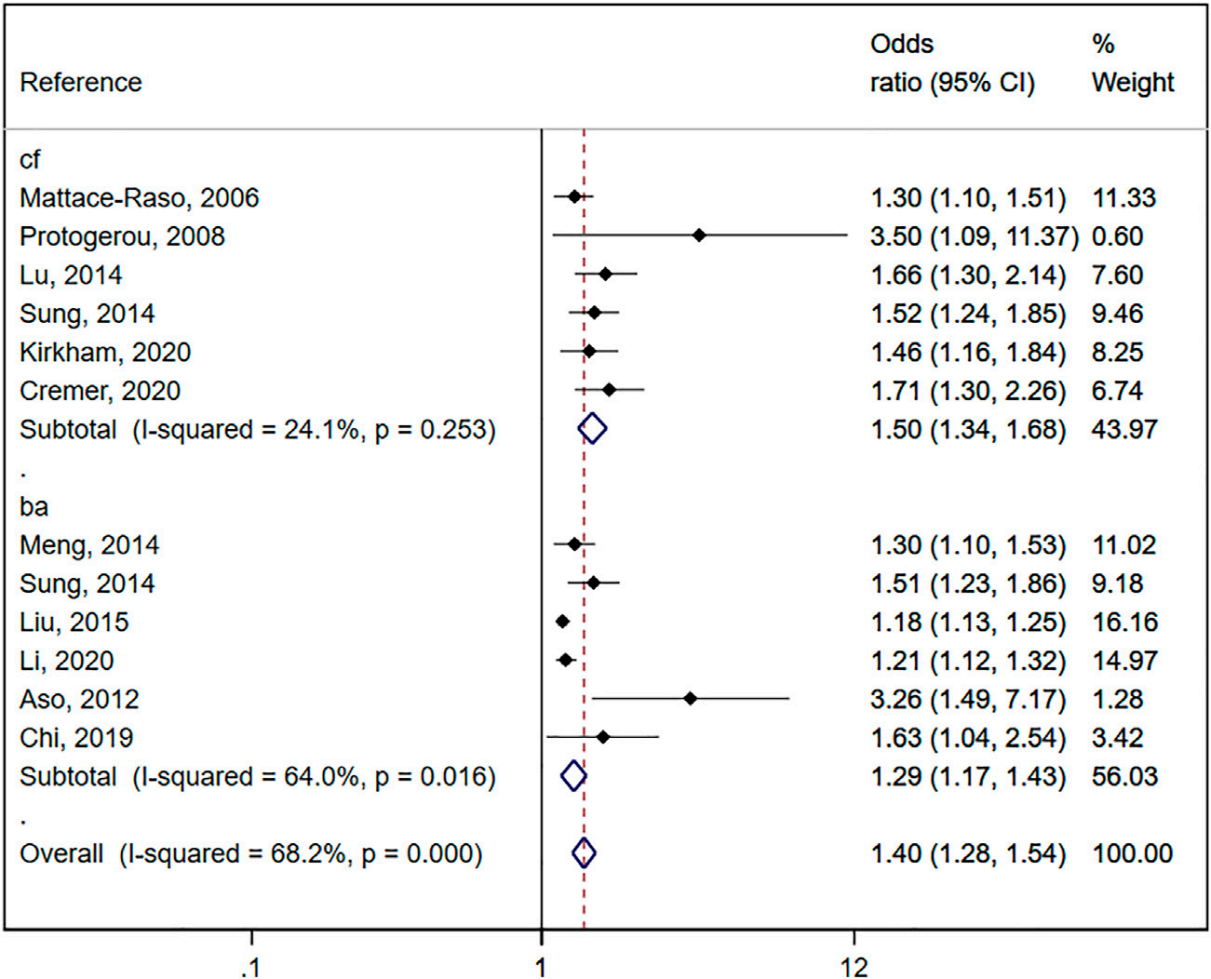
Forest plot including the association between orthostatic hypotension and arterial stiffness (central and peripheral pulse wave velocity).

Increased arterial stiffness measured by PWv was associated with an increase in the pooled OR estimate of OH (OR: 1.40; 95% CIs: 1.28–1.54). The heterogeneity of this estimate was substantial (*I* ^2^ = 68.2%; *p* = 0.000) (Figure 2).

### 3.4 Association between orthostatic hypotension and central arterial stiffness

When the association of OH and central arterial stiffness was estimated, a significant increase in subjects with OH was shown for cf.-PWv compared to subjects without OH (OR: 1.50; 95% CIs: 1.34–1.68), with no significant heterogeneity (I^2^ = 24.1%; *p* = 0.253) (Figure 2).

### 3.5 Association between orthostatic hypotension and peripheral arterial stiffness

When the association of OH and peripheral arterial stiffness was estimated, a significant increase in subjects with OH was shown for ba-PWv compared to subjects without OH (OR: 1.29; 95% CIs: 1.17–1.43), with substantial heterogeneity (I^2^ = 64.0%; *p* = 0.016) (Figure 2).

### 3.6 Sensitivity analysis

The pooled OR estimate for the association between OH and arterial stiffness was not significantly modified (in magnitude or direction) when data from individual studies were removed from the analysis one at a time.

### 3.7 Subgroup analysis and meta-regression models

Subgroup analyses were performed according to mean age (<65 or >65 years), and the pooled OR estimate showed significant results in subjects >65 years for increased arterial stiffness (OR: 1.38; 95% CIs: 1.24–1.53) (Supplementary Table S3). Random-effects meta-regression models showed that the percentage of female subjects could influence the pooled OR estimate for the association between OH and arterial stiffness (*p* = 0.028) (Supplementary Table S4).

### 3.8 Publication bias

Finally, evidence of publication bias was observed through Egger’s test for arterial stiffness (*p* = 0.000). Additionally, publication bias was found in central arterial stiffness (*p* = 0.050) (Supplementary Figure S1) and peripheral arterial stiffness (*p* = 0.003) (Supplementary Figure S2).

## 4 Discussion

This is a novel systematic review and meta-analysis to evaluate the association between OH and arterial stiffness in adults. Our findings provide a synthesis of the evidence supporting the observations that increased arterial stiffness measured by PWv is associated with a 40% higher risk of OH. Additionally, our findings show that the risk of OH is higher in central arterial stiffness (cf.-PWv) than in peripheral arterial stiffness (ba-PWv), at 50% and 29%, respectively.

Several studies have reported that OH is a predictor of cardiovascular disease (CVD) events (Rutan et al., 1992; Rose et al., 2000; Verwoert et al., 2008; Fedorowski et al., 2010; Fedorowski et al., 2011; Uraschek et al., 2018) based on the hypothesis that transient hypoperfusion of the heart by OH contributes to cumulative microvascular ischemia over time. However, this hypothesis remains controversial. (Juraschek et al., 2020). Furthermore, arterial stiffness measured by PWv has been shown to be associated with different CVD events. (Vlachopoulos et al., 2010; Ben-Shlomo et al., 2014; Zhong et al., 2018; Sequí-Domínguez et al., 2020). Our findings could show another possible hypothesis in which the mechanism by which OH could be a predictor of CVD events would be due to a continuous increase in arterial stiffness. Arterial stiffness is a chronic process (related to aging) that produces changes at both functional and structural levels and which seems to precede OH, which could be considered an acute and punctual process. Thus, episodes of OH could indicate a preclinical increase in arterial stiffness, although this possible hypothesis requires further research. However, given that a cause-effect association (similar to a chicken or egg first dilemma) cannot be established due to the nature of the study, another hypothesis to establish the association between OH and arterial stiffness in a young and healthy population could be the effect caused by orthostatic abnormalities in increasing vascular stiffness, which could evolve into clinical orthostatic disorders. (Wu et al., 2022).

Furthermore, our results showed a higher risk of OH associated with central arterial stiffness than with peripheral arterial stiffness. This could be explained by structural differences between central and peripheral arteries as central arteries have a greater number of smooth muscle cells and elastin. (Lacolley et al., 2017). The pathophysiology of OH points to alterations in smooth muscle cells and elastin (Singh et al., 2021) caused by damage or dysfunction of the baroreflex efferent pathway, which could be associated with this small difference in central and peripheral arterial stiffness (Mattace-Raso et al., 2007). Moreover, long-term increased arterial stiffness could generate functional changes in both central and peripheral arteries (Jordan et al., 2020), producing this possible association with subsequent CVD events associated with OH.

In addition to the main findings of this systematic review and meta-analysis, the meta-regression analysis showed that, in studies with larger numbers of women, the risk of OH associated with increased arterial stiffness decreased. This suggests that the underlying mechanisms of pressure-dependent arterial stiffness differ by sex. However, evidence supporting this idea is limited (Fonesca et al., 2018), and a better understanding of these interactions may be relevant for new approaches to the treatment of BP and arterial stiffness.

Some limitations to this study that could have compromised our results should be noted. First, the assessment of OH could have varied among the included studies (between 0 and 5 min). Second, there was evidence of significant publication bias with the Egger test, and the results of unpublished studies could have modified the results of our meta-analysis. Furthermore, the association between OH and arterial stiffness has only recently been investigated, and this may have been a source of publication bias. Third, the pooled OR for PWv and ba-PWv analysis showed a substantial risk of heterogeneity; therefore, our results should be interpreted with caution. Fourth, since PWv has been the accepted gold standard for non-invasive measurement of arterial stiffness (Van Bortel et al., 2011), only studies using PWv were included in this systematic review and meta-analysis. Fifth, given the scarcity of studies, it was not possible to perform subgroup analyses by age and by healthy or treated population, which are variables that may have influenced the association between OH and arterial stiffness. Finally, since the association reported by the included studies was cross-sectional in nature, a cause-effect relationship could not be inferred. Therefore, future follow-up studies should examine this cause-effect relationship between OH and arterial stiffness.

## 5 Conclusion

In summary, our data suggest that increased arterial stiffness, measured by PWv, increases the risk of OH and is higher for central arterial stiffness than peripheral arterial stiffness. Considering that OH, which is usually associated with antihypertensive, antiparkinsonian, and antidepressant treatments, has been widely associated with the risk of cardiovascular events, appropriate control of arterial stiffness could be a clinical strategy to prevent cardiovascular morbidity and mortality.

## Data Availability

The original contributions presented in the study are included in the article/Supplementary Material; further inquiries can be directed to the corresponding author.
