# Supplementary material for: Association between arterial stiffness and orthostatic hypotension: A systematic review and meta-analysis
Source: Front Physiol. 2023 Apr 28;14:1164519. doi: 10.3389/fphys.2023.1164519 (PMC10210150; doi:10.3389/fphys.2023.1164519)

Supplementary Material

Association between arterial stiffness and orthostatic hypotension: A systematic review and meta-analysis

**Alicia Saz-Lara^a^, Iván Cavero-Redondo^b*^, Vicente Martínez-Vizcaíno^a,b^, Maribel Lucerón-Lucas-Torres^a^, Carlos Pascual-Morena^a^, Irese Sequí Domínguez^a^**

*** Correspondence:** Iván Cavero Redondo, Universidad de Castilla-La Mancha, Edificio Melchor Cano, Centro de Estudios Socio-Sanitarios, Santa Teresa Jornet s/n, 16071 Cuenca, Spain.
[Ivan.Cavero@uclm.es](mailto:Ivan.Cavero@uclm.es)

# Table Supplementary 1. Search strategy for the MEDLINE database.

| 'Adults'  **OR**  'Adult population'  **OR**  'Adult subjects' | **AND** | 'Arterial stiffness'  **OR**  'Pulse wave velocity'  **OR**  'PWv'  **OR**  'Aortic stiffness' | **AND** | 'Orthostatic hypotension' |
| --- | --- | --- | --- | --- |

# Table Supplementary 2. Quality assessment with the tool for observational cohort and cross- sectional studies of the National Heart, Lung and Blood Institute for the association between orthostatic hypotension and arterial stiffness.

| **Reference** | **1** | **2** | **3** | **4** | **5** | **6** | **7** | **8** | **9** | **10** | **11** | **12** | **13** | **14** | **Quality** |
| --- | --- | --- | --- | --- | --- | --- | --- | --- | --- | --- | --- | --- | --- | --- | --- |
| Mattace-Raso et al, 2006 (4) | Y | Y | NR | Y | N | N | Y | N | Y | Y | Y | NR | Y | Y | Fair |
| Protogerou et al, 2008 (20) | Y | Y | NR | Y | N | Y | N | N | Y | Y | Y | NR | NR | Y | Fair |
| Aso et al, 2011 (13) | Y | Y | NR | Y | N | N | NA | N | Y | N | Y | NR | NR | N | Poor |
| Lu et al, 2014 (18) | Y | Y | NR | Y | N | N | NR | N | Y | N | Y | NR | NR | Y | Poor |
| Meng et al, 2014 (19) | Y | Y | NR | Y | N | N | NA | Y | Y | N | Y | NR | NR | Y | Fair |
| Sung et al, 2014 (21) | Y | Y | NR | Y | N | N | NR | N | Y | N | Y | NR | NR | Y | Poor |
| Liu et al, 2015 (17) | Y | Y | NR | Y | N | N | NA | N | Y | N | Y | NR | NR | Y | Poor |
| Chi et al, 2019 (22) | Y | Y | NR | Y | N | N | N | N | Y | N | Y | NR | Y | Y | Fair |
| Cremer et al, 2020 (14) | Y | Y | NR | Y | N | N | NA | Y | Y | Y | Y | NR | N | Y | Fair |
| Kirkham et al, 2020 (15) | Y | Y | NR | Y | N | Y | NR | N | Y | N | Y | NR | N | N | Poor |
| Li et al, 2020 (16) | Y | Y | NR | Y | N | N | NA | N | Y | N | Y | NR | NR | Y | Poor |

# 1. Was the research question or objective in this paper clearly stated?; 2. Was the study population clearly specified and defined?; 3. Was the participation rate of eligible persons at least 50%?; 4. Were all the subjects selected or recruited from the same or similar populations (including the same time period)? Were inclusion and exclusion criteria for being in the study prespecified and applied uniformly to all participants?; 5. Was a sample size justification, power description, or variance and effect estimates provided?; 6. For the analyses in this paper, were the exposure(s) of interest measured prior to the outcome(s) being measured?; 7. Was the timeframe sufficient so that one could reasonably expect to see an association between exposure and outcome if it existed?; 8. For exposures that can vary in amount or level, did the study examine different levels of the exposure as related to the outcome (e.g., categories of exposure, or exposure measured as continuous variable)?; 9. Were the exposure measures (independent variables) clearly defined, valid, reliable, and implemented consistently across all study participants?; 10. Was the exposure(s) assessed more than once over time?; 11. Were the outcome measures (dependent variables) clearly defined, valid, reliable, and implemented consistently across all study participants?; 12. Were the outcome assessors blinded to the exposure status of participants?; 13. Was loss to follow-up after baseline 20% or less?; 14. Were key potential confounding variables measured and adjusted statistically for their impact on the relationship between exposure(s) and outcome(s)?; CD, cannot determine; N: no; NA, not applicable; NR: not reported; Y: yes.

**Table Supplementary 3.** Subgroup analysis according to mean age (<65 years or > 65 years) for orthostatic hypotension.

|  | **No. studies** | **ES (95%CIs)** | **I^2^** |
| --- | --- | --- | --- |
| **Subjects >65 years** | **8** | **1.38 (1.24, 1.53)** | **60.5%** |
| **Subjects <65 years** | 3 | 1.71 (0.37, 3.05) | 27.5% |

**Table Supplementary 4.** Meta-regression according to mean age, percentage of female, smoking history, hypertension and diabetes mellitus prevalence for orthostatic hypotension.

|  | **Coefficient** | **95%CIs** | **P value** |
| --- | --- | --- | --- |
| Mean age | 0.015 | -0.014, 0.045 | 0.274 |
| % Female | **-0.008** | **-0.016, -0.001** | **0.028** |
| Smoking history | 0.006 | -0.003, 0.014 | 0.159 |
| Hypertension prevalence | 0.003 | -0.004, 0.010 | 0.351 |
| Diabetes mellitus prevalence | 0.010 | -0.007, 0.026 | 0.204 |

**Figure Supplementary 1.** Funnel plot for odds ratios of carotid to femoral pulse wave velocity.


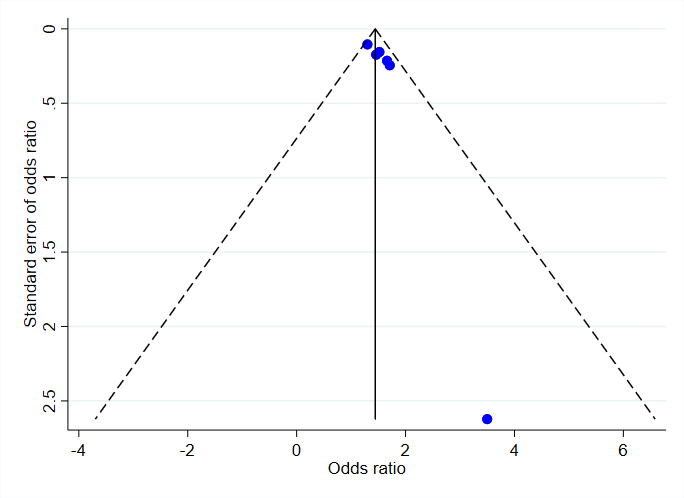


**Figure Supplementary 2.** Funnel plot for odds ratios of brachial to ankle pulse wave velocity.


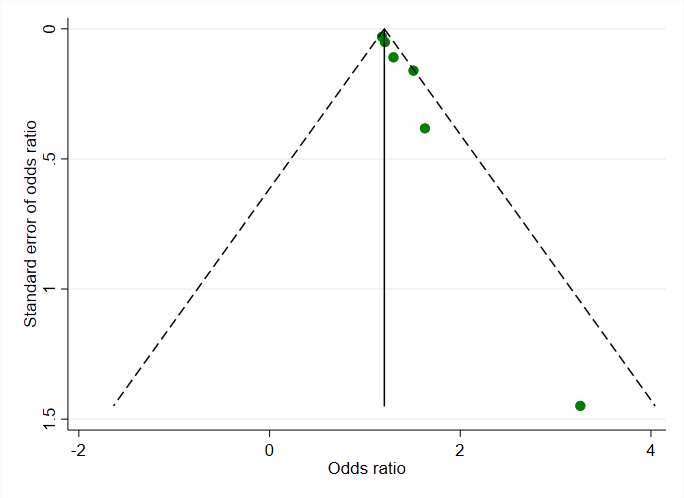

Supplement: Supplementary file 1 [file Table2.DOCX]
